# Paediatric formulations—part of the repurposing concept?

**DOI:** 10.3389/fmed.2024.1456247

**Published:** 2024-09-16

**Authors:** Siri Wang, Viviana Giannuzzi

**Affiliations:** ^1^Norwegian Medical Products Agency, Oslo, Norway; ^2^Fondazione per la Ricerca Farmacologica Gianni Benzi onlus, Research Department, Valenzano, Italy

**Keywords:** repurposing, paediatric formulations, regulatory, reformulation, market access, unmet need

## Abstract

The lack of available appropriate paediatric formulations is a significant challenge for optimal treatment in children. The resulting manipulation of adult medicines implies risk of medication errors, inaccurate dosing, and unacceptable dosage forms resulting in non-compliance. This represents significant unmet needs for a large and vulnerable patient group. Currently, the repurposing discussions seems only to a limited degree to cover the aspects of paediatric off-label use of adult medicines, including reformulation strategies to cover unmet needs for suitable formulations in the youngest age groups. Similarly, limited focus seems to be put on incentives in this specific area of repurposing. This paper will discuss the role of reformulation for paediatric needs as part of the repurposing concept, and potential factors contributing to barriers to incentivise the development of new formulations for children.

## Introduction: an important medical need

Every day, children, carers, and healthcare professionals are struggling to ensure children the care they need and deserve, namely approved and suitable forms of medicines to ensure the right dose, and safe, acceptable and effective medicines. Use of medicines outside the authorised, labelled, use (‘off-label use’) is significant in children (1). Crushing, splitting, diluting, mixing; Parents, nurses and doctors try to adapt the medicines to best possibly fit the needs of the child, whilst trying to avoid medication errors (2). This happens both for old drugs and for newly approved medicines. This lack of available appropriate paediatric formulations is a daily and significant challenge for optimal treatment in children and seems to be an important medical need (3, 4), even for essential medicines (5).

At the same time, significant focus is currently being put on fulfilling unmet needs, in particular with off-patent medicines, i.e., medicines that is no longer protected by patents. The discussion on repurposing of old medicines is part of this avenue (6, 7). The principle of targeting uncovered medical needs by use of well-known, off-patent substances by leveraging a product’s existing knowledge (on safety or efficacy) makes sense, as product development is becoming increasingly expensive, complex and with significant attrition rate.

In paediatrics, where off-label use is a particularly significant problem, repurposing represents an opportunity to generate evidence for medicines for children, to cover unmet medical needs and make paediatric drug research and development faster and easier. But is the need for age-appropriate formulations for children a part of this road? The aim of this paper is to discuss if ‘repurposing’ covers, or should cover, ‘reformulation’ in the paediatric field.

## A matter of definition?

Different *terms* are being used within this concept; repurposing, reprofiling, repositioning, and reformulation, all focusing on ‘new use’ of a product. This ‘new use’ is however not well defined: It could be for a completely different disease in another therapeutic area, a different disease within the same therapeutic area, a new route of administration (for the same or a different disease), a new treatment regimen, or a new pharmaceutical form. All these scenarios could potentially fulfil unmet medical needs, addressing off-label use, and make relevant clinical use ‘on label’, all core elements of repurposing.

Although the terms repurposing and repositioning sometimes seem to be used interchangeably (6, 7), repurposing as the overarching term is often divided into ‘repositioning’ and ‘reformulation’ (8, 9).

*Repositioning* focus on new indication for a known drug. The term ‘new indication’ seems often to be understood as within a completely different condition or therapeutic area. For example, sildenafil was originally developed for angina but was repositioned for erectile dysfunction (6), and mexiletine was originally licenced for arrythmia and repositioned for muscular disorders (10). At the same time, reprofiling/repurposing has also been described more broadly as the re-development of a drug for a ‘use that is an alternative disease or patient population than that for which it was originally developed’ (11, 12). As such, the setting with an extension of the indication to a slightly different patient cohort within the *same* disease could also in theory be covered by this definition. An expansion of the authorised age group to also include children has been specifically discussed as repositioning (13); however, there seems to be no general agreement on this.

*Reformulation* is the term used when a new formulation is being developed for an existing product. Only few papers have focused on reformulation as part of repurposing (8, 14–16) and overall, when different examples of types of repurposing are listed in the literature, reformulation as such is rarely covered. It is most often related to the setting where development focuses on a new indication (condition) but in addition a new formulation is needed, for example if the new target population is in the younger age group. The most typical example is propranolol developed for haemangioma as an oral liquid enabling dosing of the target infant population (17). Another setting is if a different route is preferred for the new indication, as in the case of minoxidil, originally developed for hypertension (oral), where the new indication (hair loss) implied both a new route (topical) and thus a new formulation (liniment) (6). Occasionally, examples are also given on solely new formulation development within the same condition/disease as the original product, e.g., venlafaxine prolonged release formulation for depression in adults (8). However, simply a new formulation, to fulfil a need for an appropriate formulation for the youngest age groups, is rarely mentioned.

A clear *definition* of the term ‘repurposing’ seems to be lacking, despite several attempts in different papers and projects. Core to them all is the overall aim to explore new uses of old substances. Whether it solely involves ‘existing products’ or also abandoned, shelved, or products under development, differs (18). Similarly, it is neither clear how far from the already authorised use this ‘new use’ should be, or, likewise, whether simply a new formulation would be captured by this term. Importantly, different stakeholders may have diverging perspectives impacting ‘their’ definition. The ‘big pharma’ understands ‘repurposing’ as the process of bringing *on-label* new therapeutic uses for already known medicines which are out of patent and regulatory data protection (19). They exclude settings like new use for drugs in development and ongoing development activities carried out by the originator, as, e.g., a coming extension of indication to children or a paediatric formulation, as agreed in a Paediatric Investigation Plan (PIP). The setting of new paediatric formulations, for off-patent products *not* under development by the originator, is not spelled out and seems neither included nor excluded.

Within the rare diseases space, increased focus is being put on drug repurposing as a way to target diseases with no therapeutic options. A recent statement argues that repurposing strategies focus on ‘new therapeutic indications for already existing drugs’ (20), but no definition is described, neither including nor excluding reformulation. The International Rare Diseases Research Consortium (IRDiRC) has developed the “Drug Repurposing Guidebook” to help developers of medicines for rare diseases to identifying tools and practises for repurposing projects (21, 22); neither here any definition of ‘repurposing’ is provided.

Notably, none of the available definitions or terms are stated by law in the EU. Notwithstanding, the Commission Expert Group on Safe and Timely Access to Medicines for Patients (STAMP) dedicated efforts to repurposing and defines drug repurposing or repositioning/re-profiling as the process of identifying a new use for an existing drug in an indication outside the scope of the original indication (23). It is also stated that it includes developing different formulations for the same drug (reformulation) (24) but does not specifically address whether a reformulation within the currently authorised disease would also be included.

In our opinion, an age-appropriate formulation, targeting a relevant off-label use in children, even for a disease or condition already authorised in adults or older children, would qualify as ‘reformulation’ within the repurposing term, It represents a new use, formally a ‘new indication’ compared to the existing label (i.e., use for an alternative patient population than that for which it was originally developed) and would ensure relevant ‘on label’ use.

## Repurposing initiatives in Europe—is reformulation part of the scope?

### Is the Paediatric Regulation taking care of formulations for paediatrics?

The focus on unmet medical needs and scientific and regulatory gaps that is essential to the repurposing concept, is in essence the rationale for the European Paediatric Regulation (25). It aims to facilitate development and availability of medicines for children by prospectively addressing medical needs in children. The focus is primarily on *on-patent* products, either on the market or to be developed. The Paediatric Regulation has actually been described as a driver for repositioning for children, in cases of deferrals which might lead to extension to paediatric indications later in time (13).

Paediatric-specific formulations are part of the requirements in this regulation (25), since products under development must be adapted to ensure that the targeted paediatric age group can be treated with safe, accurate and acceptable products. In this way, for newer products, the need for age-appropriate formulations is account for when PIPs are agreed. Although this is no guarantee that the product will actually be authorised, new formulations (e.g. added as line extensions) have been seen - following this Regulation, as described in EMA’s 10-year report to the European Commission (26). Even less a guarantee for reimbursement and marketing (27), at least there is the intention that an age-appropriate formulation must be developed for new products, if needed. For off-patent products, the Regulation introduced a dedicated – voluntary – marketing authorisation covering indications and appropriate formulations for medicines developed exclusively for children, the PUMA (Paediatric Use Marketing Authorisation). Also, research funds were allocated in the EU to develop paediatric old medicines used off-label (12).

For various reasons, the PUMA concept has been considered a limited success (26, 28). One of the main reasons is the lack of revenue, as Member States seem to recognise little added value in off-patent medicines, even if they include a new age-appropriate formulation or new paediatric indications (29). No change seems to be expected for the PUMA or paediatric-specific formulations in the coming revision of the EU General Pharmaceutical Legislation (30).

Of note, paediatric formulations for off-patent products can also be authorised via ‘hybrid applications’ or ‘well-established use’ routes (31), so outside the Paediatric Regulation, as demonstrated in a recent analysis (32). The same challenges as for PUMAs, related to lack of revenue and limited access, seem to apply also for such products.

So overall, related to formulations the Paediatric Regulation may in the long run ensure suitable formulations for the *new* products coming to the market (within the same disease area as proposed for adults), but it will not solve the continuous problem with access to appropriate formulations for *old products that are critical* for treatment of these youngest patients.

### The EMA repurposing pilot

In Europe, EMA and Heads of Medicines Agencies (HMA) have launched a pilot project to facilitate repurposing. The aim is ‘to support not-for-profit organisations and academia to gather or generate sufficient evidence on the use of an established medicine in a new indication with the view to have this new use formally authorised by a regulatory authority’ (33). The pilot is born from the above-mentioned STAMP group and focuses on well-established substances for off-patent products, and targets indications that are ‘distinct from the currently authorised ones’ (24). Thus, it seems that neither extension to a paediatric indication within the adult disease, nor simply formulations for paediatric use, is included in this pilot, despite the STAMP definition opening up for this. The criteria should be ‘likely important public health benefits’, focusing on areas where few medicines are authorised or with high morbidity/mortality despite available medicines. Although these criteria tick several of the boxes relevant for medicines for children, they are not fully clear and it is our understanding that extensions to paediatric age groups, and paediatric formulations specifically, are currently not covered by these criteria.

### Repurposing in the proposed revision of the EU pharma legislation

For the first time, a rule on repurposing could be coming in place in the EU as a whole: the proposed new EU Pharmaceutical Regulation (30) dedicates an entire article on repurposing (article 48). According to this new provision, not-for-profit entities may submit non-clinical or clinical evidence for a new therapeutic indication to the EMA. In case of a favourable opinion, the marketing authorisation holder shall submit a variation to update the product information with the new therapeutic indication. This seems to be a major step ahead in the pharmaceutical field and could have an important impact on paediatric use of medicines, as significant amount of data on off-label use could in turn become authorised. On the other hand, it is not yet clear what the term ‘new therapeutic indication’ would cover, and the area of new formulations seems not yet touched upon.

Another proposed rule could require companies to conduct paediatric studies within a PIP in a disease different from the one proposed by the developer, based on the drug’s mechanism of action. This could impose companies to also consider paediatric-specific formulations within the relevant disease.

However, a definition is still lacking here. Therefore, it is still not possible to state if, or in which cases, reformulation is a valid repurposing element. The last steps of the legislative process will result in the definitive legislative act and might guide us further on this issue.

## Discussion

### Why is reformulation rarely specified or included in repurposing concepts and projects?

Notwithstanding different *terms* are being used within the repurposing concept, to our knowledge, no definition is available by law either in Europe or in any other part of the world. Nevertheless, as in Europe, several projects/initiatives have been initiated. In the US, FDA defines drug repurposing as ‘the identification of potential novel uses of existing drugs’ (34); it includes new ways of treating diseases, new combinations of drugs, new dosing regimens and durations of therapy, new populations that can benefit from existing treatments. To note, this definition apparently comes from a collaborative FDA initiative, but not from the law. Similarly, the Australian Government has very recently (2024) introduced the Medicines Repurposing Programme (MRP) to identify new therapeutic uses for existing medicines by encouraging sponsors to expand the approved uses of their medicines and to finally have this new use approved (35), but repurposing is not defined in the Australian legislation. In our opinion, such legal or authoritative definition, regional or global, could assist the focus on the need for appropriate formulations for children, or clarify on the need for other potential incentives.

The reasons why *industry* focus has not been on simple reformulation, are most likely linked to the uncertainties regarding the expected economic revenue. As for a rare disease, the market for a paediatric-specific formulation might be limited; such formulations and/or strengths are in principle ‘orphan’ products even for non-rare diseases. Although a ‘traditional’ repositioning of an authorised product will indeed require clinical studies, the original product might be kept (33), and therefore may not require additional formulation development. Indeed, the need for a (re)formulation for a new disease has been possibly considered as one of the challenges of repurposing (22). Nevertheless, even for the same disease as originally indicated in adults, a reformulation exercise would imply a separate product in their portfolio. Uncertainty related to price and reimbursement for these new formulations will be critical and has been a recurrent discussion, both for new formulations for on-patent product lines (line extensions), but mainly so for new formulations for off-patent products (e.g., ‘well established use’-products or PUMAs). In addition, in many cases, clinical data may *still* be needed to allow authorisation in the youngest age groups. Altogether, a reformulation strategy, even within an already authorised disease, might be a challenging project embedded in uncertainties.

*Patient organizations* have had a strong voice in the increased awareness around repurposing, enforcing repurposing as a viable strategy in the relevant disease area. For example, significant input to the repurposing discussions has been provided by the rare disease community, like EURORDIS (20), or in the oncology field (36). However, the need for age-appropriate formulations as such would obviously not be covered by these stakeholders, unless part of the specific disease strategy. The paediatric population do not have the corresponding strong ‘disease-agnostic’ voice to look after their interests when comes to suitable, adapted formulations for their common diseases, despite products being off-patent and often used off-label.

Moreover, definitions might be claimed by *regulators and policy makers*, with the intention to address unmet medical needs through providing incentives and allocating funds. In this setting, it could also be a chance of intentionally restricting definitions and criteria, to enable prioritisation and ensure targeted incentives, thus impacting on research priorities and ultimately on medical needs to be covered. Clearly, the discussion on definition of repurposing (and any criteria for being considered repurposing) is becoming increasingly relevant also from an economical perspective.

### A true unmet need for paediatric formulations?

As for any repurposing project, continued off-label use of the original product is a real challenge, also for new formulations. Established practises in compounding, extemporaneous handling and manipulation may continue despite a new formulation being authorised. National policies and strategies may play an important role here, also to increase awareness of the need for authorised, appropriate formulations.

The down-stream decision-makers’ *value assessment* is probably at the core of this discussion: the added value for a new formulation will be considered as part of Health Technology Assessments and any price negotiations. Although the benefits of a paediatric-tailored formulation seem obvious, having more precise dosing, more acceptable medicines, better adherence, less medication errors, these advantages are not easily measurable. Comparative trials, in children, to give figures on these advantages are challenging, and might even have limited generalisability, e.g., if comparing to a local hospital compounded oral liquid. Price comparisons with off-label, manipulated adult products (often generics) or locally compounded products will therefore necessitate a principal view on the added value of a new formulation, both from price and reimbursement decision makers and payers.

Interestingly, when a new formulation is being developed as part of a repositioning strategy for a known substance for a new paediatric disease, it may also fulfil a wider therapeutic need. For example, propranolol is also being used in children within the ‘original’ cardiac conditions (e.g., arrythmias) for which use, including dosing, might be authorised for children, but no appropriate formulation has ever been available for the youngest. So *de facto*, provided the strength and form is considered appropriate, the reformulation for the new disease (haemangioma) could also fill the need within other conditions (despite then also being off-label).

### How to fill the gap and solve the problem?

The persistent lack of appropriate formulations is a significant challenge for children and a problem that has not yet been solved, particularly for off-patent medicines.

Any potential solutions?

- Including reformulation in the existing and coming repurposing R&D initiatives, even within an already authorised indication.
- Being clear on the definitions is considered a prerequisite, to guide funders and medicine developers. If reformulation is not considered part of the repurposing concept within the current initiatives, alternative specific R&D funding initiatives should be set up.
- Entrenchment of the importance of appropriate formulations for optimal treatment of children by all stakeholders, including policy makers, downstream decision-makers (including payers), and research funders.
- Implementing measures and incentives at regulatory level to attract manufacturers, to increase market size for these small products, e.g., English-only packages as a standard requirement.
- Improving global access to those paediatric formulations already licenced in other regions (37, 38).

In conclusion, we have to rethink the measures we take for this group of products. Including them in the repurposing concept could be a start but will not solve the problem unless other additional incentives and regulatory measures are put in place. Currently, the regulatory burden and the perceived added value does not seem to match up, potentially resulting in economically non-viable projects.

Or, do we have to give up the idea of having equally appropriate high-quality medicines for our children, and leave the challenges to children, carers, and compounding pharmacists?
